# Evaluation of a multiplex PCR screening approach to identify community-acquired bacterial co-infections in COVID-19: a multicenter prospective cohort study of the German competence network of community-acquired pneumonia (CAPNETZ)

**DOI:** 10.1007/s15010-021-01720-8

**Published:** 2021-10-23

**Authors:** Kathrin Rothe, Christoph D. Spinner, Marcus Panning, Mathias W. Pletz, Gernot Rohde, Jan Rupp, Martin Witzenrath, Johanna Erber, Frank Eberhardt, Andreas Essig, Jochen Schneider, Members of the CAPNETZ study group, M. Dreher, C Cornelissen, W. Knüppel, D. Stolz, N. Suttorp, P. Creutz, A. Mikolajewska, A. le Claire, M. Benzke, T. Bauer, D. Krieger, M. Prediger, S. Schmager, M. Kolditz, B. Schulte-Hubbert, S. Langner, O. Degen, A. Hüfner, C. Hoffmann, T. Welte, J. Freise, G. Barten-Neiner, M. Nawrocki, I. Fuge, J. Naim, W. Kröner, T. Illig, N. Klopp, C. Kroegel, A. Moeser, B. Schleenvoigt, C. Forstner, D. Drömann, P. Parschke, K. Franzen, J. Rupp, N. Käding, E. Wouters, K. Walraven, D. Braeken, C. Spinner, H. Buschmann, A. Zaruchas, T. Schaberg, I. Hering, W. Albrich, F. Waldeck, F. Rassouli, S Baldesberger, M. Panning, M. Wallner

**Affiliations:** 1 School of Medicine, Institute for Medical Microbiology, Immunology and Hygiene, Technical University of Munich, Munich, Germany; 2 Department of Internal Medicine II, School of Medicine, University Hospital rechts der isar, Technical University of Munich, Ismaninger Str. 22, 81675 Munich, Germany; 3 Faculty of Medicine, Institute of Virology, Medical Center, University of Freiburg, Freiburg, Germany; 4 Institute of Infectious Diseases and Infection Control, Jena University Hospital/Friedrich-Schiller-University Jena, Jena, Germany; 5 CAPNETZ Stiftung, Hannover, Germany; 6 Department of Respiratory Medicine, Medical Clinic I, Goethe University Hospital, Frankfurt, Germany; 7 Biomedical Research in Endstage and Obstructive Lung Disease Hannover (BREATH), Member of the German Center for Lung Research (DZL), Hannover, Germany; 8 Department of Infectious Diseases and Microbiology, University Hospital Schleswig-Holstein, Lübeck, Germany; 9 Division of Pulmonary Inflammation, Department of Infectious Diseases and Pulmonary Medicine, Charité, Universitätsmedizin Berlin, Freie Universität Berlin, Humboldt-Universität zu Berlin, Berlin, Germany; 10 Berlin Institute of Health, Berlin, Germany; 11 CAPNETZ STIFTUNG, Hannover, Germany; 12 Department of Medical Microbiology and Hygiene, University of Ulm, Ulm, Germany

**Keywords:** COVID-19, Community-acquired co-infection, Bacterial pathogens, Rapid multiplex PCR diagnostic, Antibiotic stewardship

## Abstract

**Purpose:**

Thorough knowledge of the nature and frequency of co-infections is essential to optimize treatment strategies and risk assessment in cases of coronavirus disease 2019 (COVID-19). This study aimed to evaluate the multiplex polymerase chain reaction (PCR) screening approach for community-acquired bacterial pathogens (CABPs) at hospital admission, which could facilitate identification of bacterial co-infections in hospitalized COVID-19 patients.

**Methods:**

Clinical data and biomaterials from 200 hospitalized COVID-19 patients from the observational cohort of the Competence Network for community-acquired pneumonia (CAPNETZ) prospectively recruited between March 17, 2020, and March 12, 2021 in 12 centers in Germany and Switzerland, were included in this study. Nasopharyngeal swab samples were analyzed on hospital admission using multiplex real-time reverse transcription (RT)-PCR for a broad range of CABPs.

**Results:**

In total of 200 patients *Staphylococcus aureus* (27.0%), *Haemophilus influenzae* (13.5%), *Streptococcus pneumoniae* (5.5%), *Moraxella* catarrhalis (2.5%), and *Legionella pneumophila* (1.5%) were the most frequently detected bacterial pathogens. PCR detection of bacterial pathogens correlated with purulent sputum, and showed no correlation with ICU admission, mortality, and inflammation markers. Although patients who received antimicrobial treatment were more often admitted to the ICU and had a higher mortality rate, PCR pathogen detection was not significantly related to antimicrobial treatment.

**Conclusion:**

General CABP screening using multiplex PCR with nasopharyngeal swabs may not facilitate prediction or identification of bacterial co-infections in the early phase of COVID-19-related hospitalization. Most patients with positive PCR results appear to be colonized rather than infected at that time, questioning the value of routine antibiotic treatment on admission in COVID-19 patients.

**Supplementary Information:**

The online version contains supplementary material available at 10.1007/s15010-021-01720-8.

## Introduction

Coronavirus disease 2019 (COVID-19), which is caused by the severe acute respiratory syndrome coronavirus-2 (SARS-CoV-2), emerged in China in late 2019 [1] and subsequently led to a worldwide pandemic. Although most cases of COVID-19 are characterized by mild or uncomplicated respiratory illnesses, patients may show complications such as co-infections, acute respiratory distress syndrome, and sepsis [2, 3]. Co-infections frequently occur in patients with respiratory viral illnesses such as influenza, and current guidelines recommend empirical antibiotic treatment and investigations for co-infections in patients with a severe clinical course [4–6]. For COVID-19, detailed data on community-acquired bacterial co-infections is scarce, which could be attributed to the reduced sampling frequency to reduce the risk of exposure for health care workers collecting and processing respiratory samples [7]. A co-infection can be defined as an infection occurring concurrently with the initial SARS-CoV-2 infection, while super-infections occur during the clinical course of the disease. However, these two entities are often not clearly defined in the literature, leading to varying published rates of co- and/or super-infections in patients with COVID-19.

The most frequently identified pathogens in CAP with co-infections are respiratory syncytial virus, metapneumovirus*,* influenza virus, *Staphylococcus aureus*, *Streptococcus pneumoniae*, *Haemophilus influenzae*, *Mycoplasma pneumoniae*, and *Legionella pneumophila* [8–13]*.* Previous reports have identified high rates of empirical antibiotic use, contrasting the low rates of laboratory-confirmed co-infections in COVID-19 [14–16]. For example, in one study, confirmed community-onset bacterial co-infection was reported in only 3.5% of the cases (of which 1.8% were bloodstream infections and 1.7% involved respiratory pathogens), although more than 50% of the patients were treated with empirical antibiotic therapy [17]. Overuse of antimicrobials increases the risk of adverse effects such as multidrug-resistant nosocomial secondary infections [18]. Thus, knowledge of the nature and frequency of co-infections is warranted to optimize antibiotic use in line with antibiotic stewardship principles (ABS).

Multiplex polymerase chain reaction (PCR) panels can rapidly identify the presence of relevant respiratory pathogens and may help clarify the indications for antimicrobial use as well as the choice of drugs. Therefore, this systematic prospective observational multicenter study aimed to determine whether general multiplex PCR-based screening for community-acquired bacterial pathogens (CABPs) at hospital admission could help physicians identify bacterial co-infections and predict the clinical course of COVID-19.

## Methods

### Study population

Clinical data and biological specimens from 200 patients with COVID-19 between March 17, 2020, and March 12, 2021 included in the German multicenter observational competence network for community-acquired pneumonia (CAPNETZ) [19] were evaluated in this study. 200 patients were recruited from various CAPNETZ study centers in Switzerland and in Germany: Berlin Charité Campus Benjamin Franklin (*n* = 45), Dortmund (*n* = 44), Frankfurt (*n* = 34), Lübeck (*n* = 32), Munich (*n* = 21), Bad Arolsen (*n* = 9), Gerlingen (*n* = 8), Rotenburg (*n* = 3), Basel (*n* = 2), Hannover (*n* = 1) and Bonn (*n* = 1). Every patient underwent a standardized medical assessment at the first presentation to the participating site, which included documentation of comorbidities, medical therapy, and antibiotics, details on inpatient hospital treatment, vaccination status, diagnostic procedures, laboratory parameters, and clinical symptoms. Routine microbiological work-up on admission included blood and sputum culture, pneumococcal and legionella urinary antigen. Follow-up evaluations involved assessment of structured outcome parameters at days 3, 7, and 28 after study inclusion.

### Inclusion and exclusion criteria*

The following inclusion criteria were defined:

- **Age ≥ 18 years**.
- Presentation of a new pulmonary infiltrate on chest radiography or computed tomography (CT).
- **Positive results on SARS-CoV-2 PCR testing**.
- Presence of at least one of the following clinical symptoms: cough, purulent sputum, positive auscultation, or fever.

Patients were excluded from the CAPNETZ study cohort if they met any of the following criteria:

- **Newly diagnosed active tuberculosis within the last 2 months**.
- Hospitalization > 48 h prior to inclusion.
- Noninfectious pulmonary infiltrates.

*inclusion/exclusion criteria were modified during the study period by a protocol amendment of CAPNETZ study group. Main reason for the protocol amendment was to facilitate the inclusion of patients with COVID-19. From the 13th of October 2020 onwards, only the criteria in **bold** needed to be evaluated for inclusion or exclusion of patients. 43 of 200 patients were included after the amendment of the study protocol. All patients presented clinical symptoms. 5 of 43 patients had been already hospitalized > 48 h prior to inclusion. We decided not to exclude these patients from the analysis, because SARS-CoV-2 infections were clearly community-acquired. Chest radiography/ computed tomography (CT) was either not performed (*n* = 9) or showed no pulmonary infiltrates (*n* = 19) in 28 of 43 patients.

### Sample collection

Nasopharyngeal swabs and blood samples were collected according to a standardized protocol within 48 h after hospital admission. To guarantee optimal pathogen detection quality, a standardized swab-collection and transport system for respiratory bacteria (nylon-flocked swab for nasopharyngeal collection, Product No. 503CS01, Hain Lifescience) in accordance with the CLSI standard M40-A for quality control of specimen transport devices was used. In addition, blood samples were obtained for routine clinicochemical diagnostics.

### Laboratory testing

Respiratory sample processing was performed at two clinical laboratory sites (Institute of Virology, University of Freiburg; Department of Infectious Diseases and Microbiology, Lübeck, Germany) accredited according to DIN EN ISO 15189. Samples were analyzed using a multiplex real-time RT-PCR panel previously described by Bierbaum et al. [20] to test for respiratory viruses, including adenovirus, bocavirus, coronavirus (CoV) OC43, CoV 229E, CoV HKU1, CoV NL63, enterovirus, influenza virus A + B, human metapneumonvirus, parainfluenza virus 1–4, human parechovirus, respiratory syncytial virus A + B, and rhinovirus. In addition, all samples were tested for CABPs, including *Staphylococcus aureus*, *Haemophilus influenzae*, *Streptococcus pneumoniae*, *Moraxella catarrhalis*, *Bordetella pertussis*, *Legionella pneumophila*, *Chlamydophila pneumoniae*, and *Mycoplasma pneumoniae* (ampliCube respiratory bacterial panel, Fa. Mikrogen). Laboratory confirmation of SARS-CoV-2 was performed at local centers. Blood samples were processed at the local study centers.

### Ethics statement

This study was approved by the Institutional Ethics Committee of the Hannover Medical School (Project approval number: 301-2008) and reviewed by the Institutional Ethics Committees (IECs) of all participating institutions, which operate according to the Declaration of Helsinki. Written consent was obtained from all the participants.

### Definitions and statistical analyses

Continuous data were described as median (25th and 75th percentiles) and categorical data were presented as absolute and relative frequencies. Relevant characteristics of the groups were compared using the Mann–Whitney *U* test (continuous variables) and chi-square test (categorical variables). Statistical hypothesis testing was performed using two-sided exploratory significance levels of 0.05*. Statistical analyses were performed using Microsoft Excel 2013 and IBM SPSS Statistics version 25.0 (IBM Corp, Armonk, NY). The number of patients included in the analysis was predetermined by the study protocol.

## Results

### Baseline characteristics

A total of 200 patients with confirmed SARS-CoV-2 infection were included; the median age was 58.5 years, and 63.5% of the patients were male. Cough and fever were the most frequent clinical symptoms and median oxygen saturation at admission was 91.7%. Relevant laboratory findings on admission are presented in Supplementary Table 1. While 48.0% of the patients had a preexisting cardiovascular comorbidity, 16.0% had a preexisting pulmonary comorbidity, 19.0% had diabetes mellitus, and 7.0% had a preexisting malignancy. Of these, 23.4% were admitted to intensive care units (ICUs), and of those 15.2% required mechanical ventilation. Median duration of hospital stay was 9 days and at a follow-up assessment after 3 days, 83.5% were still in hospital care, while 81.5% were discharged home 28 days after study inclusion. The 30-day mortality rate was 4.5%. Empirical antibiotic therapy was initiated in 51.5%, with ampicillin/sulbactam, ceftriaxone and piperacillin/tazobactam as the most relevant drugs. Median duration of antibiotic treatment was 5 days. In 38.8% of the patients, a combination of a beta-lactam antibiotic and a macrolide antibiotic was administered. All relevant baseline characteristics are shown in Supplementary Table 1.

### Frequency and spectrum of CABPs

In total, 43.0% of the study patients showed positive CABP PCR screening results with at least one bacterial pathogen. Of those, 17.4% presented with more than one simultaneous microbial pathogen in addition to SARS-CoV-2. *Staphylococcus aureus* (27.0%) was the predominant bacterial pathogen, followed by *Haemophilus influenzae* (13.5%), *Streptococcus pneumoniae* (5.5%), *Moraxella catarrhalis* (2.5%), and *Legionella pneumophila* (1.5%). *Mycoplasma pneumoniae*, *Chlamydophila pneumoniae*, and *Bordetella pertussis* were not detected. Findings of routine microbiological work-up on admission are illustrated in Supplementary Table 1. Positive viral PCR screening results were obtained in 1.0% of the patients, and the only detectable viral pathogen was rhinovirus.

### Comparison of COVID-19 patients with and without positive CAPB PCR screening results

Table 1 shows a comparison of patients with and without positive CAPB PCR screening results. The two groups showed no significant differences in vital signs, duration of hospital stay, levels of inflammatory markers (C-reactive protein [CRP], procalcitonin [PCT], and leucocytes), ICU admission rates, and mortality rates. Purulent sputum and higher platelet counts were associated with positive CABP PCR results. Table 2 shows a subgroup analysis of the clinical outcomes and inflammation parameters of COVID-19 patients with positive CABP PCR screening results in relation to the use of antimicrobial treatment. Patients with a positive CABP PCR screening who received antimicrobial treatment did not show better clinical outcomes for ICU admission and mortality. The duration of hospital stay was significantly longer in the group receiving antimicrobial treatment (11 days vs. 7 days; *p* < 0.01), while inflammation markers (CRP, *p* = 0.43; PCT, *p* = 0.06) did not significantly differ between the two groups (Table 2).

**Table 1:** Comparison of patients with and without positive PCR findings for respiratory bacterial pathogens

Characteristics^a^ (median [25th and 75th percentiles], unless otherwise indicated)	Total cohort	CAPB-negative findings on PCR	CAPB-positive findings on PCR	*p-*value^b^
Age (years)	58.5 (48.3–70.0)	58.5 (48.0–71.8)	58.5 (49.3–68.0)	0.67
Duration hospital stay (days) (*n* = 197)	9.0 (6.0–14.0)	9.5 (7.0–17.0)	8.0 (6.0–12.0)	0.06
Resident of long-term care facility (*n*/*N* [%])	7/200 (3.5)	6/114 (5.3)	1/86 (1.2)	0.14
30 days mortality rate (*n*/*N* [%])	9/200 (4.5)	5/114 (4.4)	4/86 (4.7)	1.00
Clinical and laboratory findings on admission				
Cough (*n*/*N* [%])	153/200 (76.5)	92/114 (80.7)	61/86 (70.9)	0.13
Purulent sputum (*n*/*N* [%])	22/200 (11.0)	8/114 (7.0)	14/86 (16.3)	0.04
Fever (*n*/*N* [%])	138/200 (69.0)	80/114(70.2)	58/86 (67.4)	0.76
Leucocyte count (G/L) (*n* = 198)	5.9 (4.8–7.7)	5.8 (4.3–7.3)	6.2 (5.1–8.1)	0.05
Thrombocyte count (G/L) (*n* = 199)	204.0 (161.0–283.0)	196.5 (158.0–251.0)	225.0 (174.0–293.0)	0.01
Lymphocyte count (/nL) (*n* = 173)	1.0 (0.7–1.4)	0.9 (0.7–1.3)	1.1 (0.8–1.4)	0.10
CRP (mg/dL) (*n* = 199)	15.1 (6.2–65.7)	16.5 (5.0–68.2)	14.3 (6.8–47.0)	0.69
CRP at d3 (*n* = 143)	3.3 (1.5–7.3)	3.0 (1.4–7.8)	3.3 (1.5–6.6)	
CRP at d7(*n* = 99)	1.9 (0.7–6.1)	2.5 (0.7–7.6)	1.7 (0.7–4.0)	
PCT (µg/L) (*n* = 162)	1.1 (0.07–0.18)	0.1 (0.07–0.17)	0.1 (0.06–20)	0.73
PCT at d3 (*n* = 100)	1.1 (0.05–0.18)	0.1 (0.05–0.19)	0.1 (0.06–0.17)	
PCT at d7 (*n* = 72)	0.09 (0.06–0.25)	0.1 (0.06–0.36)	0.08 (0.06–0.19)	
Heart rate (*n* = 199)	85.0 (76.0–96.0)	85.0 (77.0–95.0)	85.0 (76.0–99.0)	0.92
Respiratory rate (*n* = 193)	18.0 (15.0–21.0)	18.0 (15.0–20.0)	18.0 (16.0–22.0)	0.31
Temperature (*n* = 69)	37.0 (36.6–38.0)	37.0 (36.6–38.0)	37.0 (36.5–38.0)	0.83
Oxygen saturation (%) (*n* = 145)	91.7 (71.9–95.2)	91.0 (67.3–95.6)	92.2 (82.0–94.2)	0.80
Infiltrate present (*n*/*N* [%])	172/191 (90.1)	96/110 (87.3)	76/81 (93.8)	0.15
ICU admission during hospitalization (*n*/*N* [%])	46/197 (23.4)	27/112 (13.7)	19/85 (9.6)	0.87
Invasive ventilation during hospitalization (*n*/*N* [%])	7/46 (15.2)	3/27 (11.1)	4/19 (21.1)	0.42

*PCR* polymerase chain reaction, *CABP* community-acquired bacterial pathogen, *BMI* body mass index, *ICU* intensive care unit, *COPD* chronic obstructive pulmonary disease, *CRP* C-reactive protein, *PCT* procalcitonin

^a^Not all parameters were evaluated or available for all included patients, leading to differing group sizes, which are indicated by “*n*” for these variables

^b^Chi-square test or Mann–Whitney *U* test was used for comparing groups with and without positive PCR findings for CABP

**Table 2:** Comparison of clinical outcomes and inflammatory parameters in COVID-19 patients showing positive CABP PCR findings (*n* = 86) with and without antibiotic therapy

Characteristics^a^ (Median [25th and 75th percentiles], unless otherwise indicated)	COVID-19 patients showing positive CABP PCR findings	COVID-19 patients showing positive CABP PCR findings with antibiotic therapy	COVID-19 patients showing positive CABP PCR findings without antibiotic therapy	*p-*value^b^
Duration of hospitalization (days) (*n* = 85)	8.0 (6.0–12.0)	11.0 (6.5–14.5)	7.0 (6.0–9.0)	< 0.01
30-day mortality rate (*n*/*N* [%])	4/86 (4.7)	4/43 (9.3)	0/43 (0.0)	0.12
ICU admission (*n*/*N* [%])	19/85 (22.4)	13/43 (30.2)	6/42 (14.3)	0.12
Age (years)	58.5 (49.3–68.0)	61.0 (51.5–68.5)	55.0 (47.0–68.0)	0.31
Laboratory findings on admission				
CRP (mg/dL) (*n* = 86)	14.3 (6.8–47.0)	15.1 (9.2–49.1)	13.1 (6.1–46.2)	0.43
CRP at d3 (*n* = 58)	3.3 (1.5–6.6)	4.2 (1.9–7.2)	2.7 (1.1–5.4)	0.14
CRP at d7 (*n* = 39)	1.7 (0.7–4.0)	1.3 (0.7–6.0)	1.8 (1.1–3.3)	0.90
PCT (µg/L) (*n* = 70)	0.10 (0.06–0.20)	0.11 (0.07–0.25)	0.08 (0.06–0.10)	0.06
PCT at d3 (*n* = 39)	0.10 (0.06–0.17)	0.10 (0.06–0.22)	0.10 (0.06–0.13)	0.59
PCT at d7 (*n* = 29)	0.08 (0.06–0.19)	0.10 (0.06–0.28)	0.07 (0.06–0.08)	0.20
PCT ≥ 0.5 (µg/L) (*n* = 9)	5/9 (55.6)	4/9 (44.4)	0/9 (0.0)	0.21
PCT ≥ 0.25 (µg/L) (*n* = 26)	13/26 (50.0)	10/26 (38.5)	3/26 (11.5)	0.05

*PCR* polymerase chain reaction, *CABP* community-acquired bacterial pathogen, *PCT* procalcitonin, *CRP* C-reactive protein, *ICU* intensive care unit

^a^Not all parameters were evaluated or available for all included patients, leading to differing group sizes, which are indicated by “*n*” for these variables

^b^Chi-square test or Mann–Whitney *U* test comparing groups with and without antibiotic therapy

## Discussion

A better understanding of the epidemiology of bacterial co-infections in COVID-19 patients is essential for risk stratification, therapeutic strategies, and infection surveillance. Identification of the causative pathogens of potential secondary infections and the use of optimal targeted antimicrobial therapy in line with ABS principles are essential in this regard. We used a cohort of well-characterized patients with laboratory-confirmed SARS-CoV-2 infection to evaluate the value of multiplex screening PCR for CABPs in identifying bacterial co-infections in COVID-19 patients. In the current study, 43.0% of the patients showed positive CAPB PCR screening results. Other studies have reported lower bacterial pathogen yields ranging from 28.0 to 32.0% [21, 22]. We attribute the higher incidence of bacterial pathogens in the present study to the following reasons: the routine use of culture-based microbiological diagnostics from deep respiratory samples is usually restricted to ICU patients. Consequently, most of the previous COVID-19 studies analyzed ICU patients, whereas most of the patients in the current study (76.4%) were admitted in the normal wards.

The low frequency and low yield of standard culture-based microbiological diagnostics in COVID-19 patients that are not intubated and suffer from only non-productive cough raises the question of how community-acquired co-infection in the early phase of the disease can be reliably detected [16]. To address this question, a multiplex PCR panel was used instead of a culture-based microbiological approach for pathogen detection in our study. However, a major drawback of this multiplex PCR approach is the difficulty in differentiating between colonization and infection. This particularly applies to nasopharyngeal swabs, which are usually used in normal ward patients. Up to 30.0% of the human population consists of asymptomatic nasal carriers of *S. aureus* [23]. *S. pneumoniae* nasal colonization is present in 6.5% of healthy adults [24], and *H. influenzae* can be found in the nasopharynx of 20.0% of healthy adults [25]. Considering the similarities between these high colonization rates and the detection rates of the predominant pathogens in the present study (*S. aureus:* 27.0%, *S. pneumoniae:* 5.5%, *H. influenzae:* 13.5%), a high proportion of positive PCR findings obtained from nasopharyngeal swabs in this study may represent colonization rather than infection. Autopsy studies [26] have reported that potential bacterial lung super-infections occur in approximately 32% of severe COVID-19 cases. In contrast, the incidence of bacterial lung co-infection in the early phase of hospitalization is expected to be lower. Hughes et al. [15] investigated the incidence of bacterial and fungal co-infections in hospitalized COVID-19 patients and found a low frequency of bacterial co-infections in the early COVID-19 hospital presentation. Positive blood cultures were identified in 7.1% (60/836) of the patients, of which 65% were classified as contaminants. Bacteremia caused by respiratory infection was confirmed in only two cases. As demonstrated in Table 2, COVID-19 patients with positive results in CABP PCR screening who received antimicrobial treatment did not show better clinical outcomes (regarding ICU admission and mortality) than patients with positive CABP PCR findings and no antimicrobial treatment. Furthermore, the duration of hospital stay of COVID-19 patients who showed a positive CABP PCR screening result and received antimicrobial treatment was significantly longer than that of COVID-19 patients who showed positive CABP PCR results and did not receive antimicrobial therapy (11 vs. 7 days, *p* = 0.003). In summary, these findings suggest that positive CABP PCR results are not correlated with the presence of bacterial co-infections in COVID-19. Consequently, clinical correlation of positive PCR findings by using adjunctive clinical and laboratory markers is needed to determine the significance of detected pathogens. In the current study, purulent sputum and platelet counts were correlated with positive CABP PCR screening results. Nevertheless, ideal clinical or laboratory parameters to distinguish pulmonary infection from colonization still do not exist. In this context, procalcitonin (PCT) seems to be the most reliable marker [27], although PCT can neither rule out nor confirm an ongoing bacterial infection. In our study, PCT levels were not significantly higher in the positive-PCR group than in the negative-PCR group, which might be related to the immunomodulatory treatment of COVID-19 patients. Giacobbe et al. [28] compared CRP and PCT values in COVID-19 patients with bloodstream infections treated with and without immunomodulatory therapy. The median CRP and PCT values of patients receiving neither tocilizumab nor steroids were higher than those of patients treated with steroids or tocilizumab.

The multicenter design of the study adds further significance to our findings and emphasizes the importance of well-characterized cohorts and biobanks. Nevertheless, the study has several limitations: we did neither analyze asymptomatic SARS-CoV-2-negative controls nor asymptomatic SARS-CoV-2-positive controls. Compared to other COVID-19 trials, mortality rate of the current study was quite low which might be related to the young age and the low ICU admission rate of the study cohort. Due to the prospective study design, patient had to give their written consent for inclusion into the study. Furthermore, the sample size was limited; therefore, analyses of larger cohorts in further studies are warranted to clarify the value of CABP PCR screening in COVID-19.

## Conclusion

In our cohort of 200 COVID-19 patients, 43% showed a positive CABP PCR screening result within 48 h after hospital admission. Positive CABP PCR results were not associated with higher ICU or mortality rates, even in patients who did not receive antimicrobial treatment. Thus, a large proportion of CABPs detected using PCR on hospital admission may not cause secondary pulmonary infection during hospitalization. Discrimination between infection and colonization in PCR-positive patients on the basis of clinical signs or inflammation markers is difficult, particularly for S. aureus. Therefore, multiplex PCR findings obtained from nasopharyngeal swabs may not be a useful tool for streamlining antimicrobial treatment for COVID-19 patients in the early phase of hospitalization.

## Supplementary Information

Below is the link to the electronic supplementary material.

Supplementary file1 (DOCX 18 kb)

## Data Availability

All relevant data are made available in the manuscript and supplementary files.
