# Supplementary material for: Evaluation of a multiplex PCR screening approach to identify community-acquired bacterial co-infections in COVID-19: a multicenter prospective cohort study of the German competence network of community-acquired pneumonia (CAPNETZ)
Source: Infection. 2021 Oct 23;49(6):1299–306. doi: 10.1007/s15010-021-01720-8 (PMC8536912; doi:10.1007/s15010-021-01720-8)
Supplement: Supplementary file 1 — Supplementary file1 (DOCX 18 kb) [file 15010_2021_1720_MOESM1_ESM.docx]

Supplementary Table 1: Baseline characteristics of the study cohort

| Characteristics^*^ (Median [25th and 75th percentiles], unless otherwise indicated) |  |
| --- | --- |
| Age (years) | 58.5 (48.3-70.0) |
| Male sex (n/N [%]) | 127/200 (63.5) |
| BMI  n = 27 | 28.0 (26.0-34.0) |
| Duration of ICU stay (days)  n = 40 | 5.5 (3.3-11.5) |
| Duration hospital stay (days)  n = 197 | 9.0 (6.0-14.0) |
| Direct admission to hospital (n/N [%])^a^ | 197/200 (98.5) |
| Resident of long-term care facility (n/N [%]) | 7/200 (3.5) |
| Active smoking (n/N [%]) | 8/170 (4.7) |
| Previous smoking (n/N [%]) | 53/170 (31.2) |
| COPD (n/N [%]) | 5/192 (2.6) |
| Mortality rate (n/N [%]) | 9/200 (4.5) |
|  |  |
| Presence of pulmonary comorbidity (n/N [%])^b^ | 32/200 (16.0) |
| Presence of cardiovascular comorbidity (n/N [%])^c^ | 96/200 (48.0) |
| Presence of other chronic comorbidity (n/N [%])^d^ | 29/200 (14.5) |
| Presence of malignancy (n/N [%])^e^ | 14/200 (7.0) |
| Presence of diabetes mellitus (n/N [%]) | 38/200 (19.0) |
|  |  |
|  |  |
| Clinical and laboratory findings on admission  (unless otherwise specified) |  |
| Mean arterial pressure | 93.3 (86.3-101.2) |
| Cough (n/N [%]) | 153/200 (76.5) |
| Purulent sputum (n/N [%]) | 22/200 (11.0) |
| Fever (n/N [%]) | 138/200 (69.0) |
| Confusion (n/N [%]) | 5/199 (2.5) |
| Leucocyte count (G/L)  n = 198 | 5.9 (4.8-7.7) |
| Thrombocyte count (G/L)  n = 199 | 204.0 (161.0-283.0) |
| Lymphocyte count (/nL)  n = 173 | 1.0 (0.7-1.4) |
| CRP (mg/dL)  n = 199 | 15.1 (6.2-65.7) |
| PCT (µg/L)  n = 162 | 1.1 (0.07-0.18) |
| Heart rate  n = 199 | 85.0 (76.0-96.0) |
| Respiratory rate  n = 193 | 18.0 (15.0-21.0) |
| Temperature  n = 69 | 37.0 (36.6-38.0) |
| Oxygen saturation (%)  n = 145 | 91.7 (71.9-95.2) |
|  |  |
| ICU admission (n/N [%]) | 46/197 (23.4) |
| Catecholamine therapy (n/N [%]) | 11/46 (23.9) |
| Invasive ventilation (n/N [%]) | 7/46 (15.2) |
| Non-invasive ventilation (n/N [%]) | 25/46 (54.3) |
|  |  |
| (Polymicrobial) Detection of bacterial respiratory pathogen (n/N [%]) | 86/200 (43.0) |
| *-S. pneumoniae* | 11/200 (5.5) |
| *-S. aureus* | 54/200 (27.0) |
| *-H. influenzae* | 27/200 (13.5) |
| *-M. catarrhalis* | 5/200 (2.5) |
| *-L. pneumophila* | 3/200 (1.5) |
| *-M. pneumoniae* | 0/200 |
| *-B. pertussis* | 0/200 |
| *-C. pneumoniae* | 0/200 |
|  |  |
| Still in hospital at d7 (n/N [%]) | 122/200 (61.0) |
| Discharged home at d28 (n/N [%]) | 128/157 (81.5) |
| Discharged to nursing home at d28 (n/N [%]) | 4/157 (2.5) |
| Discharged to other hospital at d28 (n/N [%]) | 21/157 (13.4) |
| Discharged to other rehabilitation facility at d28 (n/N [%]) | 4/157 (2.5) |
|  |  |
| Routine microbiological work-up |  |
| Positive pneumococcal   antigen (n/N [%]) | 1/49 (2.0) |
| Positive legionella  antigen (n/N [%]) | 0/42 |
| Positive blood cultures  For CABP (n/N [%]) | 0/43 |
| Positive sputum for  CABP (n/N [%]) | 2/18 (11.1) |
| - S. aureus + E.coli | 1 |
| - S.aureus + M. catharalis + S. pneumoniae | 1 |
| Empirical antibiotic therapy (n/N [%]) | 103/200 (51.5) |
| Median duration (days) | 5.0 (3.0-7.0) |
| Ampicillin/sulbactam (n/N [%]) | 33/103 (32.0) |
| Ceftriaxone (n/N [%]) | 31/103 (30.1) |
| Piperacillin/tazobactam (n/N [%]) | 24/103 (23.3) |
| Other^f^ (n/N [%]) | 7/103 (6.8) |
| Azithromycin (n/N [%]) | 4/103 (3.9) |
| Meropenem (n/N [%]) | 3/103 (2.9) |
| Escalation of primary antibiotic therapy^g^ | 6/103 (5.8%) |

^*^Not all parameters were evaluated or available for all included patients, leading to differing group sizes, which are indicated by “n” for these variables; ^a^197 patients were hospitalized, 3 patients were discharged at the same day of hospital admission; ^b^COPD: n = 8, bronchial asthma: n = 14, pulmonary fibrosis: n = 1, sarcoidosis: n = 1, obstructive sleep apnea syndrome: n = 4, pulmonary embolism: n = 4; ^c^hypertension: n = 60, atrial fibrillation: n = 14, congestive heart failure: n = 4, myocardial infarction: n = 5, stroke: n = 6, peripheral arterial obstructive disease: n = 1, coronary artery disease: n = 6; ^d^chronic renal failure, thyroid disease, neurological disease, peripheral arterial obstructive disease, hepatic disease, gout, cholangitis, ankylosing spondylitis; ^e^breast cancer, colorectal cancer, hematologic disease, prostate cancer, lung cancer, stomach cancer, renal cell carcinoma; ^f^amoxicillin: n = 2, cefuroxime: n = 2, doxycyclin: n = 2, levofloxacin: n = 1; ^g^meropenem: n = 2, piperacillin/tazobactam: n = 1, meropenem + vancomycin: n = 2, piperacillin/tazobactam + vancomycin: n = 1. Abbreviations: BMI, body mass index; ICU, intensive care unit; COPD, chronic obstructive pulmonary disease; CRP, C-reactive protein; PCT, procalcitonin; GFR, glomerular filtration rate; CT, computed tomography.
